# Notch signaling inhibition attenuates epileptogenesis and hippocampal damage without altering mossy fiber sprouting in adolescent rats post-status epilepticus

**DOI:** 10.3389/fnmol.2026.1764473

**Published:** 2026-03-02

**Authors:** Ping Yuan, Jin Chen, Li Jiang

**Affiliations:** Department of Neurology, Children's Hospital of Chongqing Medical University, National Clinical Research Center for Children and Adolescents' Health and Disorders, Ministry of Education Key Laboratory of Child Development and Disorders, Chongqing Key Laboratory of Child Neurodevelopment and Cognitive Disorders, Chongqing, China

**Keywords:** adolescent rats, DAPT, epileptogenesis, Notch signaling, status epilepticus

## Abstract

**Background:**

Notch overactivation and aberrant neurogenesis following status epilepticus (SE) has been identified by our previous study. The current study further supplements this by exploring additional pathological changes during epileptogenesis post-SE, as well as the potential role of Notch in these processes.

**Methods:**

Rats were administered N-[N-(3,5-difluorophenacetyl)-L-alanyl)]-S-phenylglycine t-butyl ester (DAPT) immediately after SE induction. Spontaneous recurrent seizures were monitored via electroencephalogram (EEG). Hippocampal synaptic ultrastructure was analyzed using transmission electron microscopy. Nissl staining and Timm staining were performed at 28 days post-SE to evaluate neuronal loss and mossy fiber sprouting (MFS), respectively.

**Results:**

EEG recordings demonstrated that DAPT treatment significantly reduced the severity of epileptiform discharges post-SE. Transmission electron microscopy revealed decreased presynaptic active zone length and postsynaptic density thickness in the hippocampal CA1 region of DAPT-treated rats. Nissl staining indicated attenuated hippocampal neuronal loss and partial structural restoration following DAPT administration. Notably, Timm staining showed no significant effect of DAPT on MFS compared to controls.

**Conclusion:**

Inhibition of Notch signaling alleviates EEG epileptic activity, mitigates synaptic damage, and partially preserves hippocampal neuronal structure in adolescent rats post-SE, without altering MFS. These findings suggest Notch signaling as a potential therapeutic target for post-SE neuroprotection, though its role in MFS remains unclear.

## Highlights

- Pharmacological Notch inhibition significantly attenuated spontaneous recurrent seizures and associated hippocampal pathology post-SE, indicating the excessive activation of the Notch pathway accelerates epileptogenesis in the developing brain.
- This provided direct *in vivo* evidence establishing activated Notch signaling as a critical molecular driver of epileptogenesis in adolescent models, distinguishing its role from neurodevelopment.
- It identified Notch signaling as a promising, novel therapeutic target for developing disease-modifying anti-epileptogenic or anti-epileptogenic strategies following initial brain insults like SE in the juvenile period.

## Introduction

Status epilepticus (SE) or status convulsion (SC) is a neurologic emergency describing a prolonged seizure. This event can lead to neuronal death, injury, and changes in neural networks and cause cognitive dysfunction (Trinka et al., 2015). SE is accompanied by alterations of the synaptic structure and function within the hippocampus, sustained epileptic discharges, and the onset of mossy fiber sprouting (MFS). Additionally, SE induces acute increases in hippocampal cell proliferation followed by chronic depletion of neural stem/progenitor cells, as well as morphological and migratory abnormalities in granule cells—including the development of hilar basal dendrites and ectopic migration into the hilus. During SE, the number of excitatory synapses in the hippocampus increases, whereas the number of inhibitory synapses decreases (Andoh et al., 2019; Fan et al., 2023). Simultaneously, synaptic proteins and receptors involved in neurotransmitter release and synaptic plasticity are altered (Bourne and Harris, 2011). In addition, sustained epileptic discharges can propagate rapidly throughout the brain, involving multiple brain regions and disrupting normal neuronal communication, thereby leading to brain energy expenditure and metabolic disruption (Trivisano and Specchio, 2020). Notably, previous studies found that MFS can promote seizures through several mechanisms (Cavarsan et al., 2018). Abnormal mossy fibers have been linked to neosynaptogenesis on dentate granule cells, forming recurrent excitatory circuits in the hippocampus. The newly formed connections can bypass normal inhibitory control mechanisms, allowing the balance between excitation and inhibition to be disrupted (Wenzel et al., 2000; Luo et al., 2021). This further promotes seizure generation and propagation, and it is believed to play an important role in the development and maintenance of chronic epilepsy (Buckmaster et al., 2002). Additionally, hippocampal areas CA1 and CA3 are affected by epileptic activity. It has been suggested that the CA3 region can generate self-sustained epileptiform activity that can propagate to CA1, which can also provide feedback inhibition to CA3 as a regulatory mechanism to limit epileptic propagation (Huh et al., 2016). Thus, exploring the range of episodic changes in the hippocampus during sustained epilepsy is crucial for studying the generation and development of epilepsy and its underlying mechanisms.

The Notch signaling pathway plays a crucial role in neuronal development, regulating various processes such as cell fate determination, differentiation, and synaptic plasticity. Notch receptors and ligands are expressed in the developing brain, and their interactions contribute to the proper formation and function of neural circuits (Breunig et al., 2007). Abnormalities in Notch signaling components have been observed in animal models of epilepsy (Yuan et al., 2020). The Notch pathway has a pivotal role in epilepsy by influencing the differentiation, proliferation, and dendritic growth and connectivity of neurons (Yang et al., 2018; Piacentino et al., 2021). Aberrant Notch signaling can disrupt neuronal differentiation and proliferation and potentially lead to abnormal connections between neurons (Piacentino et al., 2021). Abnormal Notch signaling can affect the balance between excitation and inhibition in neural circuits, leading to hyperexcitability and seizure activity (Hortopan et al., 2010). Additionally, Notch signaling has been implicated in neuroinflammation and gliosis, which contribute to epileptogenesis and the maintenance of chronic seizures (Wu L. et al., 2018). However, the mechanism by which Notch signaling contributes to the development of epilepsy remains unclear.

We have specifically elaborated on the research regarding excessive Notch activation and abnormal neural regeneration after SE in our previous article (Yuan et al., 2020). This study serves as an additional supplement to explore other pathological changes during the process of epilepsy formation after SE and the potential role of Notch therein.

## Methods

### Rat treatment

Sprague Dawley (SD) rats were housed in an animal enclosure with a temperature of 60% humidity, a temperature of 21 ± 1 °C, and a 12 h/12 h light/dark cycle. They were fed with food and water *ad libitum*. This study was carried out in strict accordance with the recommendations in the Guide for the Care and Use of Laboratory Animals of the National Institutes of Health. The protocol was approved by the Committee on the Ethics of Animal Experiments of Chongqing Medical University (Chongqing, China). All surgery was performed under sodium pentobarbital anesthesia, and all efforts were made to minimize suffering.

SE was induced in 20-day-old male SD rats *via* intraperitoneal (i.p.) injection of pilocarpine (30 mg/kg, Sigma-Aldrich, St. Louis, MO, USA) 18–24 h after lithium chloride injection (127 mg/kg, Solarbio, Beijing, China). Each rat was randomly assigned to either the experimental group or the control group. Thirty minutes before pilocarpine, atropine (Aladdin Biochemical Technology, Shanghai, China) l mg/kg was intraperitoneally injected to reduce mortality. Rats in the experimental group were intraperitoneally injected with 10 mg/kg diazepam to terminate the seizures 60 min after SE, while rats in the control group were injected with an equal volume of sterile saline. Animals showing no seizures, subthreshold Racine grades (Racine scores <3), or seizure-related death were excluded. All behavioral seizure scoring was performed by two independent blinded observers, with discrepancies resolved by a third blinded evaluator. Seizure severity was assessed using the Racine scale, which classifies epileptic behaviors into 5 progressive stages based on motor manifestations (Racine et al., 1972). Score 1: Facial clonus (e.g., blinking, twitching of vibrissae, rhythmic jaw movements); Score 2: Head nodding accompanied by facial clonus; Score 3: Unilateral forelimb clonus with preserved posture; Score 4: Bilateral forelimb clonus and rearing (loss of balance); Score 5: Generalized tonic-clonic seizures with rearing, falling, and severe convulsions.

**Figure 1:**
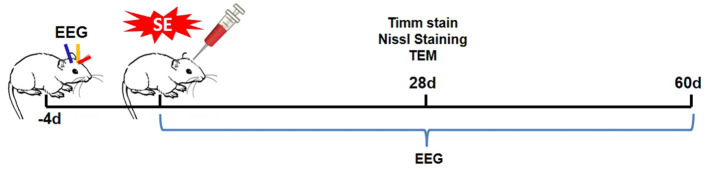
Experimental design. Rats were randomly assigned to the control, SE, or DAPT group. DAPT was administrated at 30 min after seizure termination. Brain tissues were taken 28 days after SE induction to perform Timm staining, Nissl staining, and TEM. To collect EEG data, rats in the different groups were equipped with electrodes 4 days before SE induction. EEG data were collected continuously for 60 days after SE induction (2 h per day).

Treatment groups were randomly assigned using computer-generated block randomization (stratified by litter), with cages labeled by coded identifiers to conceal group identity. 20-day-old male SD rats in the experimental group were randomly assigned to the SE or N-[N-(3,5-difluorophenacetyl)-l-alanyl)]-S-phenylglycine t-butyl ester (DAPT) group. Specifically, 5 μl of DAPT (D5942, Sigma-Aldrich) dissolved in DMSO (50 μM) were injected stereotaxically (0.5 μl/min) into the lateral ventricle of rats in the DAPT treatment group using a Hamilton syringe at 30 min after seizure termination (ether inhalation anesthesia). Stereotaxic coordinates relative to bregma were: anteroposterior (AP): −0.8 mm, mediolateral (ML): ±1.5 mm, dorsoventral (DV): −3.5 mm (based on the rat brain atlas for adolescent rodents). The needle was left in place for an additional 5 min after injection to allow for diffusion of the drug, then slowly withdrawn over 2 min to prevent backflow. Conversely, rats in the SE group only received the same volume of DMSO as the vehicle. The experimental design is presented in Figure 1.

This experiment lasted for 2 years. A total of 110 rats were used for the experiment. Analyses were performed on the same cohort of animals to enable direct structure-function correlation, with some tissues from this cohort used for other assays in the previous study (Yuan et al., 2020). Animal health and behavior were monitored once everyday. Six rats died after electrode implantation. Twenty rats died during or after SE induction, and 20 rats died during electroencephalogram examination (Table 1). Rats were euthanized 28 days after SE induction via an intracardial perfusion of sodium sulfide-containing perfusate after sodium pentobarbital anesthesia. All animal welfare considerations were taken, including efforts to minimize suffering and distress, use of analgesics. All research staff got special training in animal care or handling. Histological processing and image analysis were conducted by technicians unaware of group assignments, with slides relabeled prior to quantification.

**Table 1:** Mortality of rats in each group at different time points during the experiment.

**Time (days)**	**Control**	**SC**	**DAPT**	**Tottle**
1–4	1	2	3	6
5–7	3	9	8	20
8–28	2	7	6	15
29–60	0	2	3	5

*N*, Number of rat deaths.

Days 1–4: Post-EEG electrode implantation observation period.

Days 5–7: Post-SE model induction and intracerebroventricular injection observation.

Days 8–28: Chronic epilepsy development phase.

Days 29-60: Post-epileptogenesis monitoring period.

### Electroencephalogram (EEG) and analysis

Electroencephalogram monitoring were performed according to the previously published method (Han et al., 2021). For anesthesia, we used a ketamine-xylazine mixture (75 mg/kg + 5 mg/kg, i.p.) with supplemental doses (1/3 initial dose every 30 min) adjusted for pup weight. A hole was drilled with a diameter of 1.00 mm on each side of the sagittal suture of the rat parietal bone (*n* = 6/group), and grounded it above the telencephalon. Then a self-made simple electrode (0.1 mm nickel chromium wire) was inserted and contacted the dura mater for scalp electroencephalogram examination. The electrode assembly was secured using a combination of medical-grade dental cement and biocompatible adhesive to create a stable interface that accommodates skull growth while maintaining electrode positioning. Post-operative analgesia consisted of buprenorphine (0.1 mg/kg, s.c.) administered every 8 h for 48 h. Pups were recovered on a 32 °C heating pad for 1 h before being transferred to foster dams (acclimated to pup scent), with daily monitoring of weight and nursing behavior. For pups with feeding difficulties, we provided 0.2 mL of sterile milk formula (20% skim milk + 5% glucose/electrolytes) via gastric tube every 4 h, maintaining separation for 48 h until preoperative weight was regained. After 4 days of wound healing, induce SE according to the above method. The inclusion criteria for SE induction required: ≥5% weight gain over 24 h, normal feeding/exploratory behaviors, intact tactile responses, and absence of neurological abnormalities (e.g., seizures, ataxia) within 72 h post-op, verified through daily weighing and behavior (Table 2). During the onset of SE, perform EEG examination. Afterwards, perform 2 h (from 9:00 AM to 11:00 AM) of EEG examination every day until 60 days after SE induction. Adjust ADL instrument amplifier parameters based on previously published articles. According to a previous study (Han et al., 2021; Cao et al., 2004), seizures were defined as discharges with the following characteristics: frequency>5Hz, amplitude>2 times baseline, and duration ≥ 10s. Spike frequency amplitude, and duration were calculated by segmenting visually identified ictal epochs from 3 spontaneous seizure sessions per animal (*n* = 6 per group), averaging values across 5 consecutive spike-wave discharges per seizure, then across all seizures per session, and finally across animals to generate group-level data. The pClampfit software was used to record the average number of spontaneous seizures per day, and analyze the duration and discharge frequency of each seizure. Continuous impedance measurements and periodic visual inspections of the electrode-tissue interface were implemented, with any recordings showing impedance fluctuations >20% being excluded from analysis. The ADJUST plugin was employed to implement an automated artifact detection algorithm, which identified and excluded motion-related signals.

**Table 2:** Body weight changes and Racine scores in rats.

**No**.	**Weight1(g)**	**Weight2(g)**	**Racine1**	**Racine2**
C1	42.50	51.92	0	0
C2	43.2	52.81	0	0
C3	41.8	51.03	0	0
C4	44.00	53.83	0	0
C5	42.10	51.45	0	0
C6	43.60	53.30	0	0
SC1	41.50	50.67	4	3.69
SC2	44.50	54.42	4	4.17
SC3	42.80	52.29	3	3.49
SC4	43.00	52.53	5	1.92
SC5	41.20	50.31	5	2.89
SC6	44.8	54.80	4	3.21
DAPT1	42.30	51.70	5	1.52
DAPT2	43.41	53.05	4	2.33
DAPT3	41.00	50.02	3	2.59
DAPT4	45.01	55.03	5	1.89
DAPT5	42.03	51.30	4	2.68
DAPT6	43.92	53.62	3	2.76

C, control group; SC, SE group; DAPT, DAPT group.

Weight1, Initial body weight of 16-day-old male SD rats in each group at enrollment; Weight2, Body weight of rats in each group at 4 days Post-EEG electrode implantation; Racine1, Racine score during SE; Racine2, Mean Racine score of each rat during the chronic phase of epileptogenesis (60 days after SE).

### Timm staining to analyze MFS

Timm staining was performed using the FD Rapid TimmStain™ kit according to the manufacturer's instructions (#PK701, FD NeuroTechnology, Columbia, MD, USA). Specifically, rats were sacrificed 28 days after SE induction (*n* = 6/group) *via* an intracardial perfusion with 100 mL of 0.1 M sodium sulfide-containing perfusate, followed by 250 mL of 4% paraformaldehyde (PFA) in 0.1 M phosphate-buffered saline (PBS, pH 7.4). Brains were postfixed in 4% PFA for 24 h at 4 °C and transferred to 30% sucrose solution in 0.1 M PBS for 72 h at 4 °C. Cryoprotected brains were sectioned coronally at 40-μm intervals using a Leica CM1950 freezing microtome (Leica Biosystems, Nussloch, Germany) and mounted on positively charged slides, air-dried at room temperature for 24 h, and then stored in a light-protected box at −20 °C until staining. Under light-protected conditions, the slides were immersed in freshly prepared Timm's developer within a Coplin jar, followed by incubation in a precisely controlled 26 °C water bath for 90–150 min to allow optimal metal sulfide precipitation. Post-staining, the slides were thoroughly rinsed 3–5 times with running tap water (maintained under dark conditions to prevent photochemical artifacts), air-dried in a desiccator, and finally coverslipped with DPX neutral mounting resin for long-term preservation. Images were acquired using an Olympus BX53 microscope (Olympus, Tokyo, Japan) equipped with a 20 × objective lens. Image analysis was performed using ImageJ software (version 1.53k, National Institutes of Health, Bethesda, MD, USA). The analysis of MFS in CA3 region was performed according to the Gavazos MFS Classification Standard (0–5 scale) (Gold, 2012), where grade 0 indicates no sprouting, grade 1 represents occasional sprouting fibers in the supragranular layer, grade 2 shows a sparse but continuous band of fibers, grade 3 exhibits a dense but narrow band, grade 4 displays a dense and wide band, and grade 5 demonstrates extensive sprouting forming a dense plexus extending into the molecular layer1. The quantification was conducted by two blinded investigators who analyzed Timm-stained coronal sections of the dorsal hippocampus under light microscopy, with inter-rater reliability >90%. Only the dorsal hippocampus was analyzed in this study. The final MFS score for each animal represented the average of three consecutive sections within the dorsal hippocampal formation. In the DG region, density measurements were performed using ImageJ. The region of interest was selected in the inner molecular layer of the DG, and the integrated optical density (IOD)/area of Timm staining was calculated to reflect the sprouting intensity.

### Nissl staining

Neurons in the CA1 and CA3 regions of the dorsal hippocampus from rats in each group were analyzed by Nissl staining 28 days after SE induction (*n* = 6/group). Brain tissues were immersion-fixed in 4% paraformaldehyde (PFA) at 4 °C for 72 h. Samples were dehydrated through a graded ethanol series (70% for 10 min, 80% for 10 min, 95% for 10 min, and 100% for 15 min) followed by xylene clearing for 30 min, then embedded in high-grade paraffin wax for microtome sectioning. The paraffin sections of brain tissues after different treatments were routinely deparaffinized, hydrated through an alcohol gradient (soaked in 95%, 80%, and 70% alcohol for 1 min each). Following a 1-min rinse with distilled water, Nissl staining was performed by uniformly applying 100–200 μL of cresyl violet solution per brain section, allowing the stain to develop for approximately 3 min. Subsequently, the sections were gently rinsed under low-flow tap water for 2 min to remove excess dye. Rapid differentiation was achieved by briefly immersing the sections in 95% ethanol, followed by dehydration in absolute ethanol, clearing in xylene, and final mounting with neutral balsam. Six rats in each group were selected to generate brain tissue specimens, and five sheets of each specimen were selected for Nissl staining.

The brain slices on the cover glasses were imaged using a fluorescence microscope (IX53, Olympus) equipped with a 40 × objective lens. Positive neurons were quantified using ImageJ software (US National Institutes of Health, Bethesda, MD, USA). The analysis was performed using a semi-automated approach: images were first thresholded to identify neuronal somata, followed by manual verification to exclude non-specific staining artifacts. All analyses were conducted by an observer blinded to the experimental group assignments.

### Transmission electron microscopy

TEM was performed to observe the changes of synaptic morphology and density in the hippocampal CA1 region 28 days after SE induction in the different treatment groups (*n* = 6/group). Brain tissues were harvested immediately after transcardiac perfusion with 100 mL of 0.1 M sodium sulfide solution, followed by 250 mL of 4% paraformaldehyde (PFA) in 0.1 M phosphate-buffered saline (PBS). The perfused brains were post-fixed in the same 4% PFA solution at 4 °C for 24 h, then transferred to 30% sucrose in 0.1 M PBS for cryoprotection until they sank to the bottom. Hippocampal CA1 tissues were fixed in 2.5% glutaraldehyde for 2 h at 4 °C, washed three times with 0.1 M PBS, and stained with 2% OsO_4_ for 2 h at room temperature. After dehydration *via* a series of alcohol concentrations (30%, 50%, 70%, 80%, 95%, and 100%) and acetone, the tissue was embedded in epoxy resin to form small 5-mm spheres. Cut the embedded tissue blocks into ultra-thin sections (60 nanometers thick) using a Leica EM UC7 ultramicrotome (Leica Microsystems, Wetzlar, Germany), and stain them with uranyl acetate and lead citrate. Observation was then performed by TEM. The criteria used to identify ultrastructural synaptic profiles were previously described (Gold, 2012; Szule et al., 2015; Han et al., 2022). In order to analyze the morphological changes in the length of the presynaptic membrane active band (AD), synaptic gap (SG) width, and postsynaptic density (PSD) thickness of the hippocampus at the ultrastructural level, 5 slices were randomly selected from each sample according to the principle of systematic random sampling and photographed using TEM (magnification, × 40,000). A total of 10 micrographs were captured for each rat. The AD length, SG width, and PSD thickness were measured by hand-tracing using ImageJ. For AD length, the freehand line tool was used to trace the full linear extent of the electron-dense presynaptic active zone and measurements were recorded in nanometers (nm); for SG width, a straight line tool was applied perpendicular to the synaptic cleft at three equidistant points along the AD and the mean value was calculated; for PSD thickness, the perpendicular distance from the postsynaptic membrane to the outermost edge of the electron-dense PSD region was measured at three points and the mean value was determined for each synapse.

### Statistical analysis

All data were recorded as the mean ± SD and statistically analyzed using SPSS 17.0 software (IBM, Armonk, NY, USA). An unpaired Student's *t*-test was used for comparisons between two groups. For multiple comparisons, one-way analysis of variance with Dunnett's T3 test was used. All datasets were assessed for normality using Shapiro-Wilk tests (α = 0.05) and homogeneity of variance with Levene's test. Parametric tests (ANOVA/*t*-test) were applied only when both assumptions were satisfied; otherwise, Mann-Whitney U test for independent groups was used. *p* < 0.05 was considered statistically significant.

### Data availability statement

All relevant data generated in this research can be requested from the corresponding author.

## Results

### Effect of Notch signaling on epileptic discharges after SE

Our system demonstrated stable recording capabilities throughout the 2-month period, as evidenced by consistent electrode-skin interfacial impedance values (maintained within 15% of baseline) and reproducible signal characteristics across recording sessions. The inhibitory effect of DAPT on the Notch signaling pathway has been experimentally validated through Western blot and immunofluorescence assays, as demonstrated in the authors' previously published work (Yuan et al., 2020).

**Figure 2:**
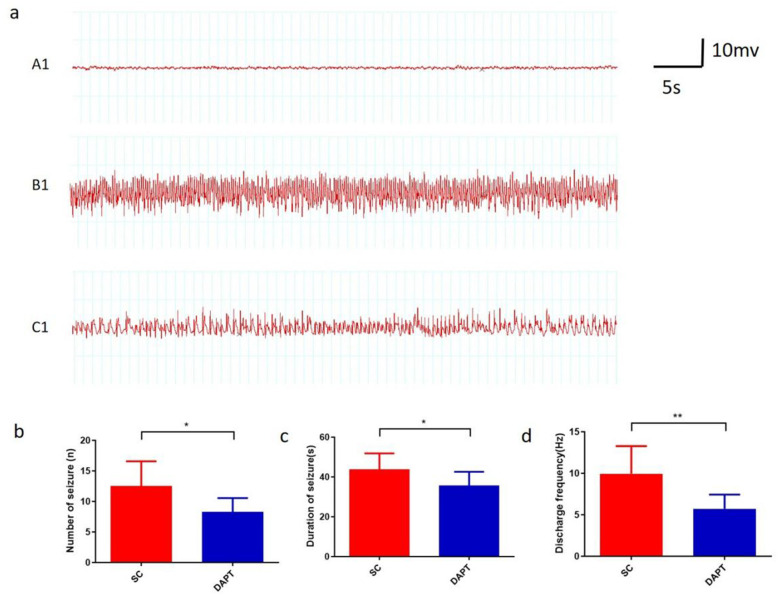
EEG recordings of rats from different groups. **(a)** EEG recording of SRS in different groups. (A1) Control group. (B1)SRSs in the SE group. (C1) SRSs in the DAPT group. **(b)** number of spontaneous seizures per day. **(c)** duration of SRS. **(d)** discharge frequency of each seizure.*p < 0.05, **p < 0.01. *n* = 6/group.

Epileptogenesis is a pathological process in which sporadic recurrent seizures (SRSs) gradually appear after SE. Rats in both the SE and DAPT groups could detect varying degrees of SRS during the chronic phase of epileptogenesis afer SE, manifested as scattered spike and spike waves lasting for tens of seconds (Figure 2a). Frequent epileptiform discharges occurred in the acute phase (1–3 days) after SE induction. The number of epileptiform discharges increased slightly over time, but the duration of each epileptiform discharge shortened. By contrast, although SRSs also appeared in the chronic period after SE induction in the DAPT group, the amplitude of the sharp and spiky waves decreased, and the frequency of the discharges slowed. In DAPT group, the average number of spontaneous seizures per day [12.55 ± 1.12 vs. 8.29 ± 0.86, *p* = 0.019, 95%CI (0.77, 7.76), Figure 2b], duration of seizure [43.83 ± 2.24 vs. 35.77 ± 2.59, *p* = 0.038, 95%CI (0.49, 15.62), Figure 2c] and discharge frequency of each seizure [9.94 ± 0.93 vs. 5.73 ± 0.65, *p* = 0.006, 95%CI (1.36, 7.07), Figure 2d] were significantly lower compared with that in SE group. Moreover, the degree of epileptic seizure behavior in the DAPT group rats was significantly reduced compared to the SE group (Table 2). In the DAPT group, seizures in rats were mainly characterized by binocular gaze and facial muscle twitching. In the SE group, rats typically exhibit seizure behavior with unilateral or bilateral forelimb spasms. No statistically significant difference was observed in the latency period from acute SE to the onset of SRS between the SE group and the DAPT-treated group, indicating that DAPT intervention did not affect the latency to spontaneous epileptic seizures. These results suggest that Notch signaling blockade after SE induction partially attenuates the severity of EEG epileptic discharges.

### Effect of Notch signaling on the synaptic ultrastructure in the CA1 region of the hippocampus after SE induction

**Figure 3:**
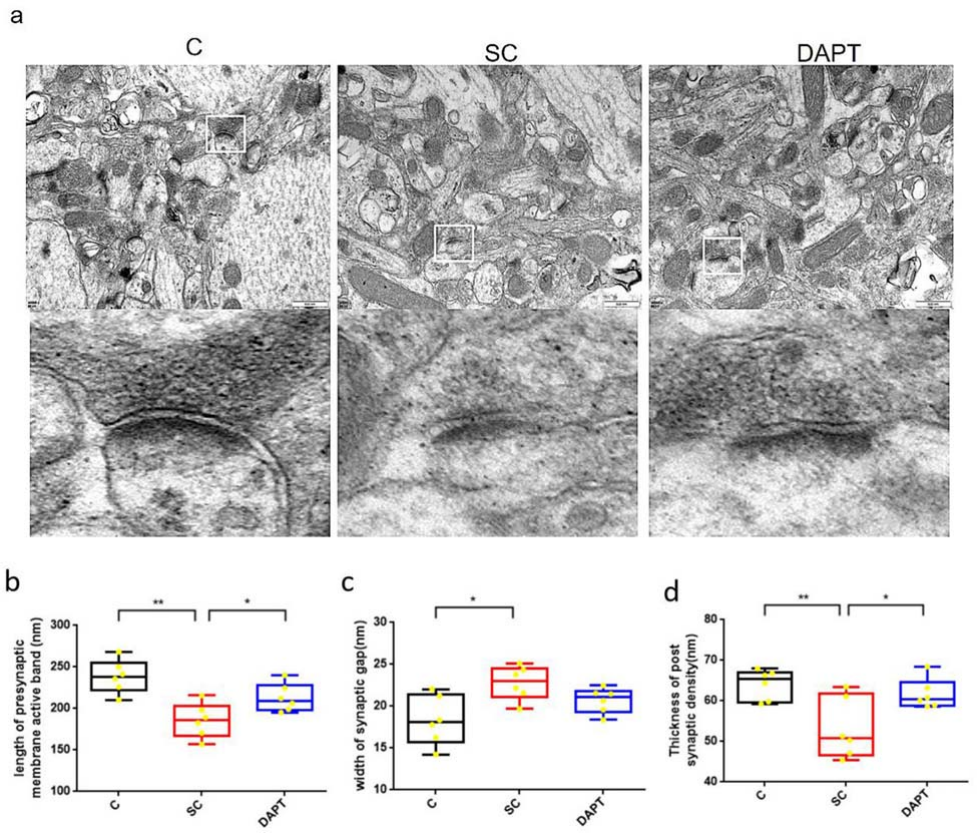
Changes in the hippocampal synaptic ultrastructure after SE induction. **(a)** TEM images of the hippocampal CA1 region on day 28 in the Control, SE, and DAPT groups. Scale bars: 500 nm. **(b–d)**: Indicators of the hippocampal synaptic ultrastructure in the different groups. Comparison of the AD length in brain slices **(b)**, SG width **(c)**, and PSD thickness **(d)** among the control, SE, and DAPT groups in the chronic phase. *p < 0.05, **p < 0.01. *n* = 6/group. C, control group; SC, SE group; DAPT, DAPT group.

The presynaptic membrane active band is the specialized site for neurotransmitter release where synaptic vesicles dock and fuse. Its structural integrity is critical for regulating vesicle release probability and location, which directly impacts synaptic transmission reliability and neural coding (Rozenfeld et al., 2023). The SG width influences the spatiotemporal dynamics of neurotransmitter diffusion and receptor activation. Pathological cleft expansion may decouple pre- and postsynaptic signaling (Jiang et al., 2024). The PSD contains scaffold proteins that organize glutamate receptors and signaling complexes. Its thickness reflects the density of these molecular machines, which determines synaptic strength and plasticity (Heo et al., 2023). Reduced PSD thickness correlates with diminished excitatory postsynaptic currents and cognitive deficits. The effects of Notch signaling blockade on the synaptic ultrastructure of the CA1 region after SE induction were investigated by TEM (Figure 3a). The AD length, SG width, and PSD thickness were measured in each group. The results demonstrated that in the SE group the AD length was significantly reduced [238.6 ± 8.14 vs. 185.5 ± 8.44, *p* = 0.0011, 95%CI [(6.99, 79.24), Figure 3b], the SG was widened [18.32 ± 1.21 vs. 22.78 ± 0.83, *p* = 0.0121, 95%CI (−7.71, −1.21), Figure 3c], and the PSD was blurred compared with the control group. In addition, the thickness of the PSD was reduced [64.08 ± 1.15 vs. 53.10 ± 3.05, *p* = 0.0091, 95%CI (3.39, 18.58), Figure 3d]. By contrast, DAPT administration partially reversed the reductions of the AD length [185.5 ± 8.44 vs. 212.9±6.87, *p* = 0.0306, 95%CI (−51.62, −3.14), Figure 3b]and PSD thickness [53.10 ± 3.05 vs. 61.67 ± 1.51, *p* = 0.0306, 95%CI (−16.16, −0.98), Figure 3d], but it had no significant effect on the SG width (Figure 3c). This suggests that inhibiting the activation of Notch signaling after SE induction can promote the repair of the synaptic ultrastructure.

### Effects of Notch signaling on hippocampal CA3 and CA1 pyramidal neurons after SE induction

**Figure 4:**
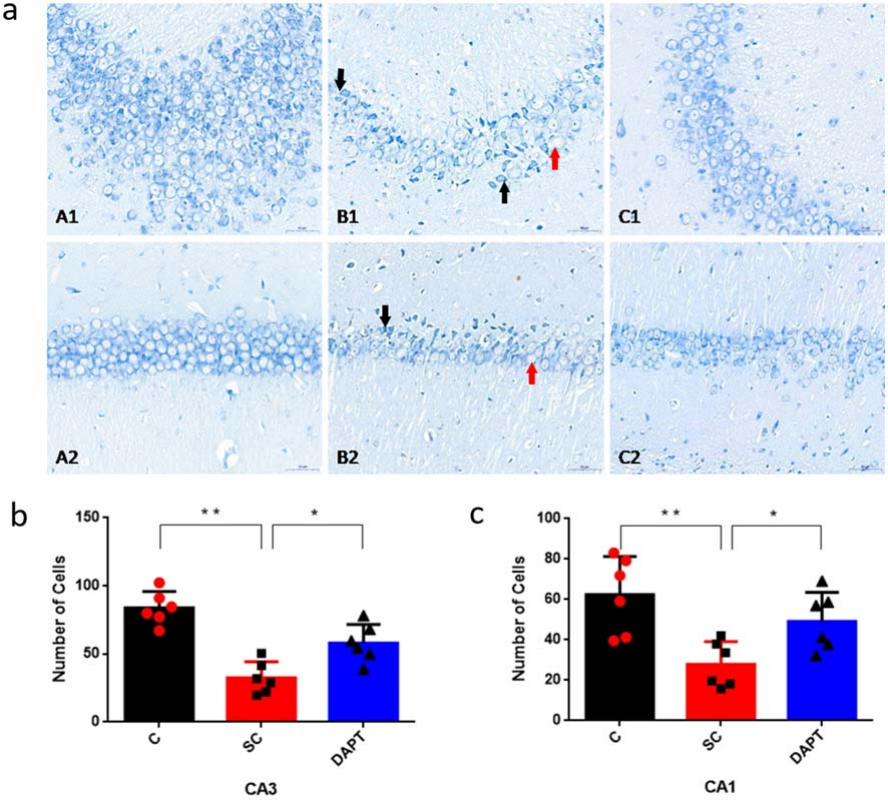
Dynamics of hippocampal CA3 and CA1 pyramidal neurons after SE induction. **(a)** Nissl staining of the hippocampal CA3 (A1–C1) and CA1 regions (A2–C2) 28 days after SE induction: A1/A2, control group; B1/B2, SE group; C1/C2, DAPT group. Black arrows indicate cell wrinkles; red arrows indicate cellular vacuolization. Scale bar: 50 μm. **(b)** Comparison of the number of Nissl-stained cells in the CA3 region: bar graph “C” corresponds to panel A1, “SC” to B1, and “DAPT” to C1. *p < 0.05, **p < 0.01. **(c)** Comparison of the number of Nissl-stained cells in the CA1 region: bar graph “C” corresponds to panel A2, “SC” to B2, and “DAPT” to C2. *p < 0.05, **p < 0.01. *n* = 6/group. Individual data points represent mean values per animal, error bars denote SD. C, control group; SC, SE group; DAPT, DAPT group.

The structure and damage of hippocampal CA3 and CA1 pyramidal neurons in each group were examined by Nissl staining 28 days after SE induction. The results showed that the arrangement of hippocampal CA3 pyramidal neurons in the SE group was loose, and some cells exhibited wrinkles or vacuolization (Figure 4a). Notably, compared with the control group findings, there were fewer Nissl bodies in the pyramidal cells of the CA3 [32.50 ± 4.85 vs. 83.78 ± 4.92, *p* < 0.0001, 95%CI (−66.60, −39.95), Figure 4b] and CA1 [27.90 ± 4.62 vs. 62.40 ± 7.70, *p* = 0.0033, 95%CI (−54.51, −14.48), Figure 4c] regions of the hippocampus in the SE group, and these findings were accompanied by scattered nucleolysis and disorganized cell arrangement. After DAPT administration, the disorganization of neuronal cells in the CA1 and CA3 regions was significantly improved, and the cellular hierarchy was distinct. The cell size and morphology were basically normalized (Figure 4a), and the number of homogeneously stained Nissl granules in the cytoplasm was increased in the CA3 (57.99 ± 5.60 vs. 32.50 ± 4.80, *p* = 0.0062, 95%CI (15.62, 35.36), Figure 4b] and CA1 (49.19 ± 5.85 vs. 27.90 ± 4.62, *p* = 0.017, 95%CI (12.4, 30.2), Figure 4c] regions compared with SE groups. This suggests that after SE induction, the inhibition of Notch signaling by DAPT partially reduced the occurrence of neuronal loss in hippocampal CA3 and CA1 region.

### Effect of Notch signaling on MFS after SE induction

**Figure 5:**
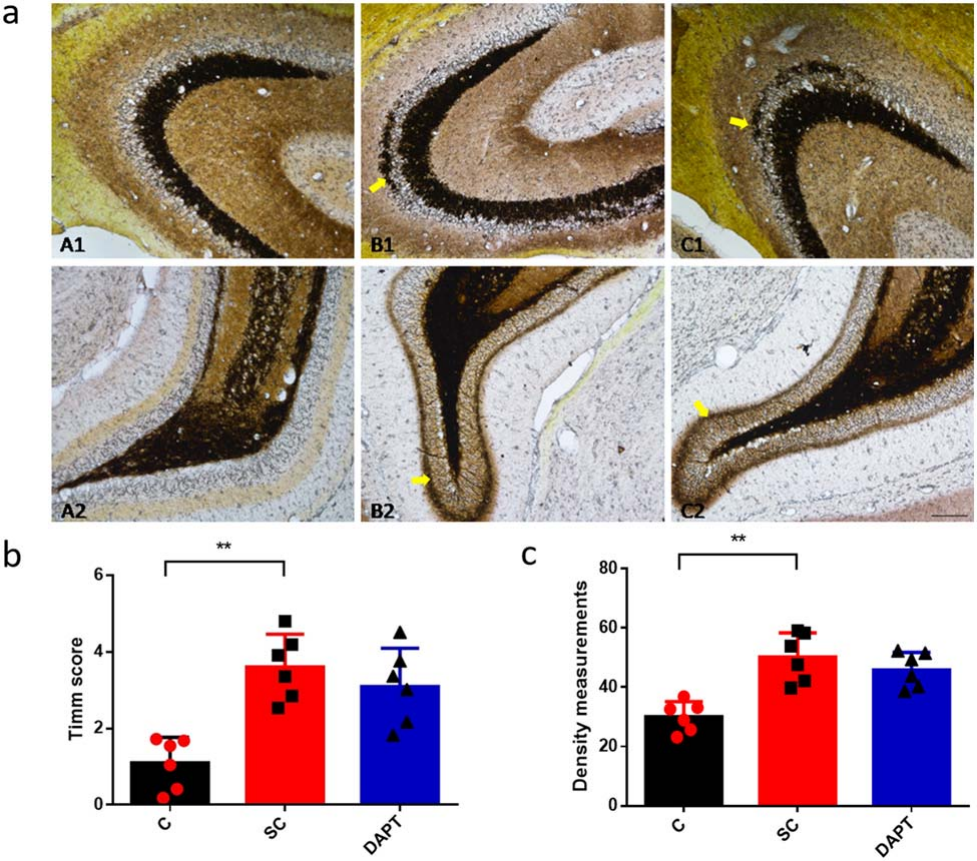
MFS after SE induction. **(a)** Timm staining of the hippocampal CA3 (A1–C1) and DG regions (A2–C2) in the control, SE, and DAPT groups 28 days after SE induction: A1/A2, control group; B1/B2, SE group; C1/C2, DAPT group. Yellow arrows indicate the quantified regions in the hippocampus. The region of interest was selected in the inner molecular layer of the DG. Scale bar: 50 μm. **(b)** Comparison of the number of Timm scores in the CA3 region: bar graph “C” corresponds to panel A1, “SC” to B1, and “DAPT” to C1. *p < 0.05, **p < 0.01. **(c)** Comparison of the MFS density in the DG region: bar graph “C” corresponds to panel A2, “SC” to B2, and “DAPT” to C2. **p < 0.01. *n* = 6/group. Individual data points represent mean values per animal, error bars denote SD. C, control group; SC, SE group; DAPT, DAPT group.

Timm staining was applied to observe changes in the distribution and number of Timm-stained granules to characterize the distribution of MFS and synaptogenesis. Timm scores and MFS densities were examined on the slices of hippocampal regions from different groups. The Timm scores in the hippocampal CA3 region was higher than that in the control group [3.61 ± 0.35 vs. 1.10 ± 0.28, *p* = 0.0002, 95%CI (1.52, 3.49), Figure 5b], which also exhibited a significantly higher number of Timm-stained particles (Figure 5a) and MFS density in the DG region was significantly increased in the SE group [49.19 ± 5.85 vs. 27.90 ± 4.62, *p* = 0.017, 95%CI (12.4, 30.2), Figure 5c], In the DAPT group, both the Timm scores in the CA3 region and MFS densities in the DG region were significantly increased compared to the control group, but no significant difference was observed when compared to the SE group, indicating that DAPT treatment failed to improve MFS after SE induction.

## Discussion

Adolescence is a critical period for hippocampal neurodevelopment, including synaptic pruning, myelination, and maturation of glutamatergic/GABAergic circuits. These processes are highly vulnerable to epileptic insults, making adolescent models more relevant to pediatric epilepsy, where SE often occurs during neurodevelopmental windows. In our study, we employed multiple complementary techniques to examine different aspects of hippocampal circuitry after SE in adolescence and the role of the Notch pathway in epileptogenesis. It revealed that inhibiting Notch signaling with DAPT during the acute phase following seizures effectively reduced the susceptibility to epileptic seizures in the chronic phase in rats, decreasing both the frequency and severity of seizures, in line with previous findings (Wu L. et al., 2018; Vezzani et al., 2011; Liu et al., 2014). SRS is a key EEG detection indicator for epileptogenesis after SE (Löscher et al., 2017). In this study, DAPT treatment significantly attenuated the severity of epileptic episodes, though it failed to fully suppress SRSs. All of these findings highlight the complexity of the development of epilepsy as a multifactorial process. This research illustrated the potential role of the Notch signaling pathway in epilepsy.

The hippocampal neural circuit plays a crucial role in the onset of epilepsy, with abnormal synaptic connections affecting the transmission of information between neurons (Greenberg and Pal, 2007). This includes both excessive strengthening and weakening of synaptic connections, as well as alterations in pre-synaptic and post-synaptic neurons, leading to instability within the neural network (Yingjun et al., 2017). Previous research suggested that abnormal changes in the synaptic structure can lead to synaptic reorganization, triggering new neural circuits that enhance synchronous firing and excitability, ultimately precipitating epileptic seizures (Engel and Pitkänen, 2020). In recent years, the pivotal role of the Notch signaling pathway in modulating the morphology and function of synapses has received increasing attention (Billiard et al., 2012). In this study, DAPT was utilized to inhibit Notch signaling, observing notable increases in AD length and PSD thickness. This suggests that inhibiting the Notch signaling pathway contributes to the partial reverse of synaptic structures. However, our synaptic ultrastructural analysis was confined to the hippocampal CA1 region, excluding DG-CA3 synapses or perforant pathways more directly implicated in epileptogenesis, so observed CA1 synaptic changes may reflect downstream effects rather than causal mechanisms of network activity alterations. Meanwhile, we only quantified AD length and PSD thickness, lacking synapse count and density data, and without complementary electrophysiological or functional validation. In future studies, we will address these gaps by extending our analysis to key epileptogenic circuits including the dentate gyrus-CA3 axis and perforant pathway inputs. We will also incorporate complementary functional measures, such as electrophysiological assessments of synaptic efficacy, excitation-inhibition balance, and network synchronization, to definitively determine whether Notch inhibition promotes synaptic functional restoration or enhances excitatory drive.

The neuronal structure in different hippocampal regions might have distinct roles in different stages of epileptic seizures. For example, once epilepsy occurs, abnormal electrical activity in the CA3 region can rapidly spread to other regions, including CA1, giving rise to the characteristics of generalized epilepsy (Kim, 2016). In this process, the CA1 region serves as an integrator and transmitter of information, receiving inputs from the CA3 region and other sources and transmitting them to other brain areas, thereby driving the further spread of epilepsy (Kim, 2016; Palacio et al., 2017). Additionally, the CA1 region is closely associated with learning and memory (Wu Z. et al., 2018), and this region might be disrupted during epileptic states, further highlighting its multifaceted role in epilepsy. The results of Nissl suggested that inhibiting Notch signaling with DAPT after SE induction partially reduced neuronal loss in both the CA1 and CA3 regions. These findings align with related research (Yoon et al., 2012; Zhang et al., 2015; Liu et al., 2021), emphasizing the potential role of the Notch signaling pathway in epilepsy development.

MFS is one of the most extensively studied pathological mechanisms associated with epilepsy (Song et al., 2015; Wang et al., 2018). However, whether MFS is “epileptogenic” or “restorative” remains controversial (Cavarsan et al., 2018). Although the reorganization of hippocampal circuits may be the cause of hippocampal epileptiform activity, recent studies have shown that the development of MFS can be independent of the of mossy fiber target loss (Cavarsan et al., 2018; Ad et al., 2002). It exists in animals with spontaneous seizures, but its presence may not be directly related to the occurrence of spontaneous seizures (Cavarsan et al., 2018; Nissinen et al., 2001). Adult born granulosa cells form functional recurrent synapses, which make a strong contribution to MFS (Cavarsan et al., 2018; Hendricks et al., 2017). It has been proven that although granulocytes born after SE can produce morphological changes in MFS, these synapses cannot drive recurrent excitation (Cavarsan et al., 2018; Hendricks et al., 2017). Therefore, MFS may not be epileptic or antiepileptic, and it can be considered as an accompanying phenomenon related to epilepsy (Cavarsan et al., 2018). Interestingly, in the present study, inhibition of the Notch signaling pathway by DAPT did not significantly reduce Timm scores and MFS density, suggesting that Notch signaling block after SE may not attenuate chronic phase convulsions by affecting MFS. We must acknowledge the limitations in methods and technology. Timm-based measurements alone cannot provide definitive volumetric data or discriminate between specific types of axonal reorganization, emphasizing the need for complementary methods such as unbiased stereology in the future when precise circuit-level changes must be quantified.

Notch signaling may serve as a pleiotropic regulatory hub, playing a central role in epileptogenesis. In previous studies (Yuan et al., 2020), increased proliferation of neural stem cells in the hippocampus of immature rats during the acute phase after SE was found to coincide with elevated Notch1 expression. DAPT reduces the number of stem cells during the acute phase, suggesting that Notch signaling may be involved in the regulation of neurogenesis after SE. Additional studies suggest that inhibition of Notch signaling may alter the expression of synaptic proteins (e.g., STX7), which regulate neuronal excitability and synchronized activity in epilepsy models (Cavarsan et al., 2018). Acute inhibition of STAT3 phosphorylation prevents the ‘imprinting' of epileptic activity patterns, loss of GABAergic neurons, and sustained reactive gliosis (Cavarsan et al., 2018), suggesting that Notch may influence neural network synchronization via the JAK/STAT pathway. In CDKL5 deficiency models (Cavarsan et al., 2018), TrkB signaling-mediated epileptogenesis can be suppressed by reducing TrkB activity, implying that Notch might regulate synaptic activity through neurotrophic factor receptors. This study further corroborates the relationship between Notch signaling and epilepsy.

The current study has several important limitations that should be acknowledged. First, this study exclusively utilized young male rats during adolescence, potentially limiting generalizability to female subjects and other developmental stages. Second, the pharmacological induction method (LiCl-Pilo) represents just one experimental approach among several established models, each with distinct advantages. The kainic acid (KA) model offers alternative seizure induction pathways, while electrical stimulation models better mimic clinical neuromodulation therapies. Third, the study employed wild-type rats rather than transgenic animals with spontaneous seizures, which could provide more clinically relevant insights into genetic epilepsies. Additionally, while the LiCl-Pilo model effectively induces status epilepticus, it may not fully recapitulate human epileptogenesis processes observed in developmental or genetic epilepsy models. These methodological constraints highlight the need for future studies incorporating diverse models, sexes, and age groups to enhance translational relevance.

## Conclusion

In summary, this study provided crucial insights into the mechanisms by which the Notch signaling pathway participates in the pathogenesis of epilepsy. By examining various aspects of the impact of this pathway, including its effects on the frequency and severity of seizures, synaptic structure, neuronal structure, and MFS, we obtained a more comprehensive understanding of its potential roles. However, it is essential to acknowledge that the functions of this pathway are diverse, and they might vary because of individual differences. Therefore, future research should seek to increase understanding of this pathway to facilitate the development of individualized treatments for epilepsy.

## Data Availability

The original contributions presented in the study are included in the article/supplementary material, further inquiries can be directed to the corresponding author.
